# Immunoglobulin Gene Repertoire in Chronic Lymphocytic Leukemia: Insight into Antigen Selection and Microenvironmental Interactions

**DOI:** 10.4084/MJHID.2012.052

**Published:** 2012-08-09

**Authors:** Efterpi Kostareli, Maria Gounari, Andreas Agathangelidis, Kostas Stamatopoulos

**Affiliations:** 1 Hematology Department and HCT Unit, G. Papanicolaou Hospital, Thessaloniki, Greece; 2 Institute of Agrobiotechnology, Center for Research and Technology Hellas, Thessaloniki, Greece; 3 Department of Genetics, Development and Molecular Biology, School of Biology, Aristotle University of Thessaloniki, Thessaloniki, Greece; 4 Laboratory of B cell Neoplasia and Unit of Lymphoid Malignancies, Division of Molecular Oncology, Department of Onco-Hematology and MAGIC (Microenvironment and Genes in Cancers of the blood) Interdivisional Research Program, Istituto Scientifico San Raffaele and Universita Vita-Salute San Raffaele, Milano, Italy

## Abstract

Immunogenetic analysis of the B cell receptors (BCRs) has been a richly rewarding field for unraveling the pathogenesis of human lymphomas, including CLL. A biased immunoglobulin gene repertoire is seen as evidence for selection of CLL progenitor cells by antigen. Additional corroborative evidence is provided by the differential prognosis of cases with distinct mutational status of the clonotypic BCRs. However, perhaps the strongest immunogenetic evidence for the importance of interactions with microenvironment in driving CLL development and evolution is the existence of subsets of patients with quasi-identical, stereotyped BCRs, collectively accounting for a remarkable one-third of the entire cohort. These observations have been instrumental in shaping the notion that CLL ontogeny is functionally driven and dynamic, rather than a simple stochastic process. From a clinical perspective, ample evidence indicates that immunogenetic information can be used for the biologically and clinically rational categorization of CLL, with important potential implications for basic, translational and clinical research.

## Introduction

Chronic lymphocytic leukemia (CLL) is a disease of aged populations and the most common adult leukemia in the Western world. It is a chronic, incurable disease and very heterogeneous in terms of response to treatment: in fact, some patients reach complete and prolonged remissions, while others relapse early and need several lines of treatment.^1^ This clinical heterogeneity is linked to and likely reflects the underlying molecular and cellular heterogeneity of the disease.^2^

Several lines of research have demonstrated beyond doubt that CLL can be subdivided into subgroups with distinct biological features, extending from genomic aberrations^3^ to immune signaling^4^ via receptor molecules of both innate (e.g. Toll-like receptors) and adaptive nature (i.e. the B cell receptor, BCR), with the latter so far thought to play perhaps a more pivotal role.^2^

The importance of antigenic stimulation through the BCR in CLL development and evolution is evidenced by: (i) restrictions in the immunoglobulin heavy variable (*IGHV*) gene repertoire expressed by the clonotypic BCRs; (ii) different prognosis of patients with different *IGHV* gene mutational status; and, (iii) the existence of subsets of patients sharing BCRs with restricted, quasi-identical immunoglobulin sequences (stereotyped BCRs).

With the widespread application of assays for the determination of *IGHV* gene mutational status in a clinical context, a large amount of information became available for both biological investigations and prognostication. Thanks to these and other developments, CLL is now considered as the prototype for cancers where micro environmental interactions are critical in the onset, expansion and progression of the disease.^2^

## Origins of Immunoglobulin Diversity

B cells are key players in adaptive immune responses and crucial in the recognition and elimination of both exogenous as well as autologous threats to homeostasis.^5^ This fundamental function of B cells is dependent on a two-step process, where the creation of diverse antigen receptors represents the first step and selection by antigen the second. Indeed, the immune system relies on the formation of an extremely diverse population of B cells, each expressing on its surface numerous identical immunoglobulin (IG) molecules, which serve as the antigen receptors for adaptive immune responses (B-cell receptor, BCR).

Each IG molecule is composed of four polypeptide chains; two identical heavy chains (HC) and two identical light chains (LC).^6^ Each IG chain can be subdivided into a variable (V) and a constant (C) region. The V region is the part of the molecule that recognizes antigen, while the C region has effector functions. Each V region comprises of four areas of relatively limited diversity, known as the framework regions (FRs), interspersed with three hypervariable regions, known as complementarity determining regions (CDRs), which confer the IG molecule its unique specificity.

BCR diversity rests on the combined effect of molecular events taking place during B cell maturation. First, the random assembly of one each of multiple distinct variable (V), diversity [(D) – for IG heavy chains only] and joining (J) genes, referred to as combinatorial diversity, leads to a huge variety of combinations and corresponding molecular structures.^7^,^8^ A significant boost in diversity emerges during V(D)J recombination, since (i) nucleotides may be trimmed from the ends of the recombining genes; and, (ii) random nucleotides may be added at the V-D, D-J or V-J junctions located within CDR3, the most diverse part of the V region (“junctional diversity”). Just considering the combinatorial events of the heavy and light chain gene loci, there are greater than 1.6 x 10^6^ possible combinations for IG BCR.

In later phases of B cell ontogeny, diversity is exponentially increased as a result of further modifications by the somatic hypermutation (SHM) and class-switch recombination (CSR) processes, both driven by antigen encounter and orchestrated by an enzymatic activity called activation-induced cytidine deaminase (AID).^9^ SHM is characterized by the introduction of mutations within rearranged genes, which increases antibody diversity and produces antibodies with higher specificity.^10^ CSR replaces the constant (*IGHC*) gene to be expressed from *IGHM* to *IGHG* or *IGHE* or *IGHA*, switching antibody production from IgM to a different class, such as IgG, IgE or IgA, without changing antigen specificity.^11^ SHM and CSR have been estimated to increase the potential for variation 10^3^–10^6^ fold. Hence, altogether, the B-cell repertoire comprises of, in principle, 10^12^ different specificities. In other words, the probability that two independent B-cell clones carry exactly the same IG BCR by chance alone is virtually negligible (10^–12^).

The unique IG BCR expressed by each B cell clone can be viewed as its molecular ‘identity’. Therefore, it is no surprise that the study of immunoglobulin (IG) genes has been instrumental in understanding immune physiology and pathology. In fact, immunogenetics has provided an upgraded perception into both the ontogenetic derivation of B cell malignancies and the possible involvement of interactions with antigens in their onset and evolution.^12^,^13^ In this respect, CLL is a model disease for showing how IG gene analysis can assist in understanding the processes underlying lymphomagenesis.^2^

## Immunoglobulin Genes in CLL: Early Days

The immunogenetic history of CLL can be traced back to the 1990s when pioneer studies reported restrictions in immunoglobulin heavy variable (*IGHV*) gene usage^14^,^15^ and distinctive antigen-binding sites among unrelated cases;^16^,^17^ however, the series were small, thus hindering definitive conclusions. A major step forward occurred in 1998, when Chiorazzi’s group published the first comprehensive study confirming that the *IGHV* gene repertoire of CLL is restricted and also different from that of normal IgM^+^ B cells, with certain genes, such as *IGHV1-69*, *IGHV4-34*, and *IGHV3-7*, clearly over-represented in CLL.^18^ Pronounced restrictions were also recognized regarding the usage of *IGHD* and *IGHJ* genes. Indeed, only five *IGHD* genes were used by almost half of CLL cases, with the *IGHD3-3* gene being the most frequent. Also, the repertoire of *IGHJ* genes was characterized by predominance of the *IGHJ4* and *IGHJ6* genes. Focusing on rearrangements of the predominant *IGHV* genes, specific associations were identified with certain *IGHD* and *IGHJ* genes, best depicted by rearrangements utilizing the *IGHV1-69* gene, which were strongly biased toward the usage of the *IGHD3-3* and the *IGHJ6* genes. On the contrary, weaker or no biases were noted for cases expressing other *IGHV* genes, particularly *IGHV4-34*, *IGHV3-7* and *IGHV3-23*. Furthermore, the imprint of SHM was not uniform among *IGHV* genes: for example, the *IGHV1-69* gene carried very few or no mutations as opposed to the *IGHV3-7*, *IGHV3-23* and *IGHV4-34* genes, which were significantly mutated.^18^ These biases in the IG gene repertoire were justifiably taken as evidence for the implication of antigens in CLL development, thus seriously challenging the then prevailing concept of CLL as a neoplasm of antigen-naïve B cells.

With hindsight, the turning point in the immunogenetic research of CLL came in 1999 when the Hamblin and Stevenson and Chiorazzi groups independently demonstrated that the mutational status of the rearranged *IGHV* genes directly correlated with patient survival.^19^,^20^ As a general principle, patients carrying mutated *IGHV* genes generally follow a more indolent course than those with unmutated *IGHV* genes, who tend to show evidence of advanced, progressive disease, adverse cytogenetic profiles, clonal evolution, and resistance to therapy.

## Immunoglobulin Genes in CLL: Coming of Age, Alias Prognostication and Beyond

The load of somatic mutations across the sequence of the rearranged *IGHV* genes was the first highly accurate molecular marker for disease prognostication and remains one of the strongest independent prognostic markers in CLL. Importantly, it is independent of the actual tumor burden and also does not change during the clinical course. With the realization of its potential clinical utility came a flurry of sequencing activity focused on the IG genes: it was reassuring that all studies corroborated the general rule: “unmutated-bad prognosis, mutated-good prognosis”.

Yet, this general rule could not be applied in all cases, as first shown by Rosenquist’s group who reported that usage of the *IGHV3-21* gene in CLL IG BCRs may represent an adverse prognostic factor, regardless of the actual SHM load.^21^ Another open issue concerns the usage of a cut-off value for assigning a case to the mutated or unmutated category. In particular, both pioneering studies on the prognostic impact of IGHV gene mutational status have suggested a cut-off value of 98% identity with the closest germline *IGHV* gene as the most reliable discriminator between unmutated (≥98%) and mutated cases (<98%).^19^,^20^ This choice was necessitated by practical considerations since in 1999 the complete sequence of the human genome was not available, therefore, in principle, the observed differences from the germline could represent polymorphisms rather than somatic mutations. The cut-off value was a short cut to exclude potential polymorphic variant sequences, thus bypassing the need to sequence the corresponding germline gene in each patient.^22^

The 98% identity cut-off value has been confirmed by all subsequent studies and is still considered the best, though approximate, discriminator for clinical prognostication. That notwithstanding, one has to keep in mind that this cut-off is statistically rather than biologically relevant:^23^ in other words, it has very questionable utility for purposes other than clinical prognostication, especially in view of the fact that even a low level of mutations can be functionally relevant.^24^ Furthermore, even when used for prognostication, caution is clearly recommended for cases of “borderline” SHM status, which have been reported to comprise of a mixture of benign and malignant cases rather than a homogeneous group with moderate malignancy.^25^

A final parameter to be taken into account is the differential impact of SHM in rearrangements utilizing different *IGHV* genes, first noted by Chiorazzi’s group^18^ and subsequently confirmed in a large study by our group where we showed that the *IGHV* gene repertoires of subgroups of cases with <98% (“mutated”), 98-98.9% (“borderline mutated”), 99-99.9% (“minimally mutated”) or 100% identity to the germline (“truly unmutated”) differed significantly.^26^ On these grounds, we suggested that the presence of little or no mutations in CLL IG BCRs should not be viewed as a marker of naivety rather it could reflect selective pressures for maintaining the germline configuration.

The accumulation of IG BCR sequence data brought unprecedented surprises. For instance, following traditional immunological thinking, no-one could have anticipated that approximately half of CLL cases utilizing the *IGHV3-21* gene carry distinctive IGs with highly restricted VH CDR3s and biased pairing with lambda light chains utilizing the *IGLV3-21* gene.^27^ Considering how remote the chances are that this might happen by chance alone, this finding was rightly considered as evidence for common antigenic drive, perhaps of pathogenic significance.

## Stereotyped BCRs: CLL Development is not Stochastic

A peculiar finding in early immunogenetic studies of the IG repertoire in CLL was that unrelated CLL cases could carry highly similar VH CDR3s characterized by shared amino acid motifs.^16^,^17^ This was confirmed by the landmark study by Chiorazzi’s group, who reported the existence of subsets of BCR IGs with highly restricted VH CDR3s and proposed that a common antigen could be selecting out clones eventually leading to the observed restrictions.^28^

With hindsight, this aspect of CLL immunobiology remained of somewhat marginal interest until the *IGHV3-21* story broke the news. However, rather than a noteworthy exception, it turned out that sequence restriction was rather common as it was reported independently by different groups in both Europe and the US.^29^,^30^ Interestingly, even in these initial, rather small-scale studies (compared to what would follow –see below), certain themes emerged: homologous BCRs were present among both mutated and unmutated cases; different types of homologous BCRs existed, enabling the grouping of different cases into subsets based on common sequence features of the BCR IG, in particular the VH CDR3. These highly similar BCRs were referred to as “stereotyped”,^28^ clearly fulfilling the definition of stereotype as “something conforming to a fixed or general pattern”.

Right from the start, stereotyped BCR IGs were seen as the strongest immunogenetic evidence that selection rather than serendipity drives CLL development. Several aspects and implications of stereotypy, however, remained to be elucidated.

## BCR Stereotypy: How to Identify it and How Frequent is it?

In all studies of BCR stereotypy, the focus is on the IG heavy chains, in particular the VH CDR3, on the grounds that the more similar the primary VH CDR3 sequences of two IGs, the more similar their folding and, perhaps, their specificities.^31^

The first set of criteria for the definition of subsets with stereotyped IGHV/IGHD/IGHJ rearrangements was proposed in 2004 by Chiorazzi’s group:^28^

- usage of the same IGHV/IGHD/IGHJ germline genes,
- usage of the same IGHD gene reading frame, and
- VH CDR3 amino acid identity ≥60%, in line with established bioinformatics concepts for evaluating sequence conservation in protein sequences (e.g. amino acid substitution matrices such as BLOSUM62).^32^

In 2007, we modified the clustering algorithm allowing sequences to be clustered together even when their *IGHV* genes differed, provided the criterion of VH CDR3 sequence conservation was met.^33^ The validity of this approach is exemplified by a cluster now defined as subset #1, defined by usage of the *IGHD6-19* gene in reading frame 3 and the *IGHJ4* gene in association with different *IGHV* genes (namely *IGHV1-2, IGHV1-3, IGHV1-18, IGHV1-8, IGHV5-a, IGHV5-51, IGHV7-4-1*). Interestingly, all these *IGHV* genes are members of the same *IGHV* phylogenetic clan,^34^,^35^ i.e. they are phylogenetically linked and carry related germline sequences, thus they can produce overall homologous VH domains when recombining with identical *IGHD* and *IGHJ* genes. Subset #1 is not a conglomeration of sequences simply happening to look similar: independent studies have demonstrated beyond doubt that the cases assigned to subset #1 have similarities extending from primary IG gene sequences to genomic, functional and clinical features.^26^,^33^,^36^-^38^

**Figure 1:**
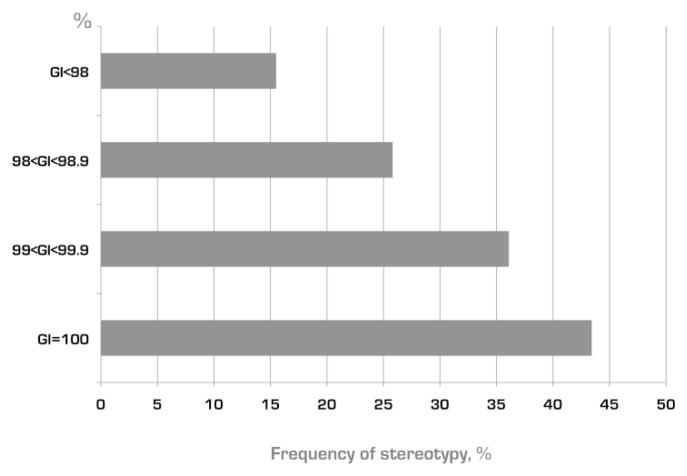
Frequency of BCR IG stereotypy among subgroups of rearrangements of different mutational status Almost half of truly unmutated rearrangements (100% identity to the germline, GI) are assigned to subsets with stereotyped VH CDR3 regions, while the frequency of stereotypy showed a trend toward decrease when the mutational load of the rearrangements was increasing. Based on data from Agathangelidis et al. 2012.^47^

**Figure 2:**
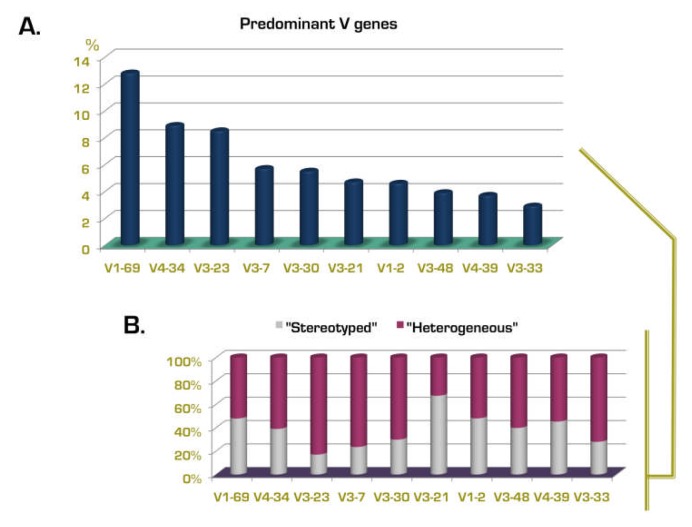
IGHV gene repertoire in CLL and relation to BCR IG stereotypy Relative frequency (%) of the 10 predominant IGHV genes in CLL (A), and their distribution among the “stereotyped” and “heterogeneous” subsets (B). The majority of *IGHV3-21* cases carry stereotyped B cell receptors, whereas most rearrangements of the *IGHV3-23* gene exhibit heterogeneity within their VH CDR3s. Based on data from Agathangelidis et al. 2012.^47^

The main findings of studies based on this clustering approach, which are still valid today, can be summarized as follows:

1. BCR IG stereotypes exist among both mutated and unmutated CLL, though significantly more frequently in the latter (Figure 1).
2. different versions of BCR IG stereotypes can be defined based on shared VH CDR3 amino acid sequence patterns which are distinct for each subset; accordingly, there are many different subsets with distinct stereotyped BCRs.
3. the relative size of each subset can differ markedly, from just a pair to large numbers of cases with homologous BCRs; indeed, certain major subsets are remarkably populated - e.g. subset #1 mentioned above and subset #2 (utilizing the IGHV3-21 gene) each account for ~2.5% of the entire cohort!^26^,^36^
4. different genes show a markedly different “propensity” to be used in stereotyped rearrangements in CLL (Figure 2) – in other words, the frequency of stereotyped rearrangements exceeded 30% in cases using certain *IGHV* genes (e.g. *IGHV3-21, IGHV1-69, IGHV1-2, IGHV1-3, IGHV4-39, IGHV3-48*), whereas it was very low (<5%) for other IGHV genes (e.g. *IGHV3-7, IGHV3-74*).^33^
5. subsets with stereotyped VH CDR3 are often characterized by restricted IG light chain gene usage and CDR3 features.^33^,^39^,^40^
6. selected subsets may be associated with distinctive clinical/phenotypic features or outcome (see following section), raising the possibility that a particular antigen binding site can be critical in determining clinical presentation and possibly also prognosis.^33^,^36^
7. the frequency of BCR IG stereotypy in CLL can exceed 25% or one-fourth of the entire cohort.

All these observations spurred great enthusiasm and, at the same time, also raised many important questions. It was reasonably felt that the observed sequence restrictions in CLL could reflect a corresponding restriction also in terms of the selecting antigens. Not paradoxically, the quest for antigens^41^-^45^ went along with the quest for subsets. However, the progressively increasing amount of IG sequence information rendered the previously used methods for identifying BCR IG stereotypy problematic as they lacked efficiency, robustness, and, importantly, sensitivity.

In order to overcome these limitations, we developed purpose-built bioinformatics methods based on sequence pattern discovery, enabling the reliable identification of VH CDR3 sequence similarities regardless of the IGHV/IGHD/IGHJ genes used. We first applied this approach in a study of 2662 patients with CLL where we reported that CLL actually consists of two different categories, based on the BCR repertoire, with important biological and ontogenetic differences.^46^ The first includes cases with heterogeneous BCRs (non-clustered cases), while the second (almost 30% of cases) is characterized by a remarkably high frequency of BCR stereotypy (clustered cases).

A pivotal aspect of this new approach was the ability to recognize more distant relationships between sequences, which can form the basis for the discovery of subsets at successive hierarchically higher levels, characterized by more broadly shared sequence patterns and, hence, greater size. Intriguingly, high-level clusters were found to be still characterized by striking *IGHV* repertoire restrictions, with only six *IGHV* genes (*IGHV1-69, IGHV1-3, IGHV1-2, IGHV3-21, IGHV4-34, IGHV4-39*) accounting for >80% of cases.^46^ A corollary of this finding is that the IG gene repertoire restrictions reported as typical of CLL are in essence a property of the category of clustered cases with stereotyped IG BCRs, whereas non-clustered cases exhibit a much less skewed repertoire.

The analysis of the IG repertoire in CLL, including BCR stereotypy, recently culminated in a study of 7596 IG VH (IGHV-IGHD-IGHJ) sequences from 7424 CLL patients, three times the size of the largest published series.^47^ In that study, we used an updated version of our purpose-built clustering algorithm, now requiring that only sequences carrying *IGHV* genes of the same clan can be assigned to the same subset, and also adopted more stringent criteria, including the requirement for identical VH CDR3 lengths and identical sequence pattern offsets (i.e. exact locations within the VH CDR3 region) between connected sequences.

**Figure 3:**
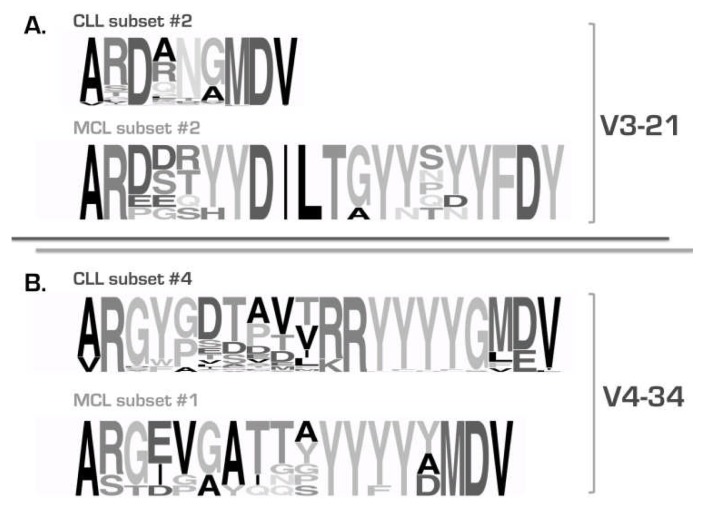
CLL stereotypes are “disease-specific” The comparative analysis of VH CDR3 sequences in CLL showed that they were clearly distinct, in terms of amino acid composition and VH CDR3 length, from those identified in MCL carrying the same *IGHV* genes: *IGHV3-21* (A) and *IGHV4-34* (B). The height of symbols within the stack indicates the relative frequency of each amino or nucleic acid at that position. Modified from Agathangelidis et al. 2012.^47^

**Figure 4:**
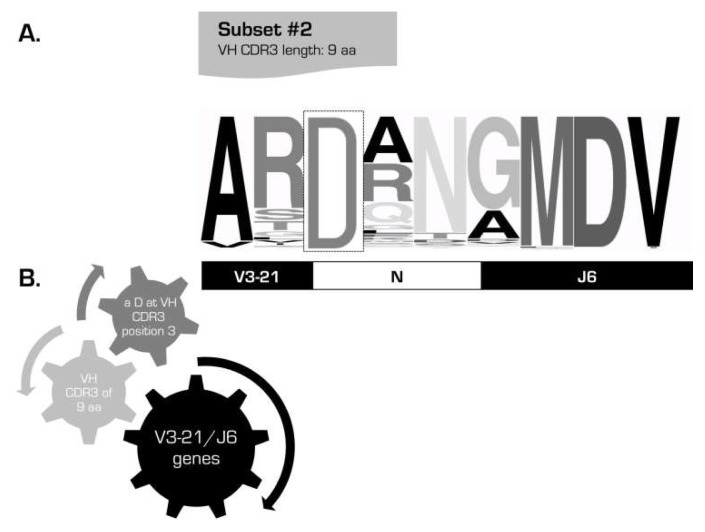
Subset #2: a unique length and a single VG CDR3 residue define a subset Sequence logo of the VH CDR3 region of cases belonging to subset #2, one of the largest subsets in CLL. The height of symbols within the stack indicates the relative frequency of each amino or nucleic acid at that position (A). Set of criteria required for the assignment of rearrangements to this particular subset (B). Rearrangements assigned to this subset can be simply identified by the usage of the IGHV3-21 gene, a VH CDR3 of 9 amino acids (aa), and an acidic residue (D) at the third position of the VH CDR3.

**Figure 5:**
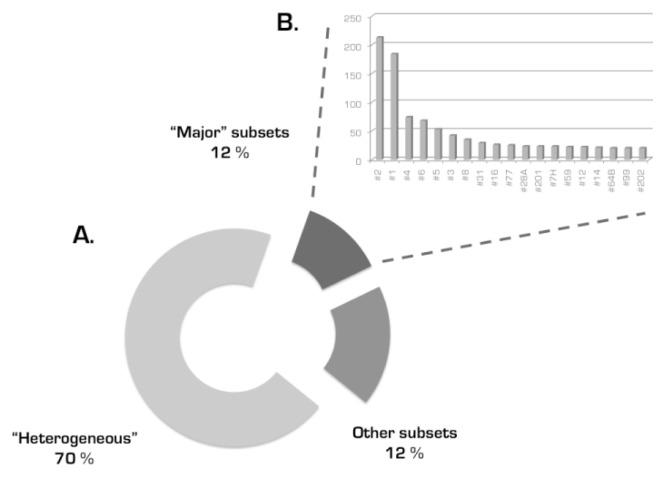
A significant fraction of the entire CLL cohort is represented by a limited number of VH CDR3 stereotypes Altogether, these “major” subsets accounted for 12% of cases in the recent study by *Agathangelidis et al* (A). The magnitude of these subsets ranged from 20 to 213 sequences (B).^47^

This analysis provided strong evidence to our previous claim^46^ that CLL indeed comprises two distinct categories, one with stereotyped and the other with heterogeneous IG, in an approximate ratio of 1:2. Furthermore, it offered answers to several questions regarding BCR stereotypy:

1. the existence of subsets of cases with stereotyped BCRs expressing different yet phylogenetically related *IGHV* genes is a consistent feature of the CLL IG repertoire: in addition to the well-established subset #1 (*see above*), several other examples came to the fore, including subsets #12 (*IGHV1-2* and *IGHV1-46*), #59 (*IGHV1-58* and *IGHV1-69*) and #77 (*IGHV4-4* and *IGHV4-59*). Therefore, it is possible that related genes might be favored for selection by particular antigens perhaps because specificity is endowed by only a few critical *IGHV*-encoded residues in association with a distinctive VH CDR3
2. not all CLL will end up belonging to stereotyped subsets, even if the cohort size increases significantly, as also supported by random simulations
3. the BCR IG stereotypes in CLL are fundamentally different from those recently reported in other B cell malignancies (Figure 3), e.g. splenic marginal-zone lymphoma^48^,^49^ and mantle cell lymphoma,^50^ alluding to distinct, disease-biased selective and ontogenetic processes
4. the sequence patterns defining subsets can be broadly divided into two types: (i) “mainly combinatorial”, i.e. largely encoded by the germline sequences of the D-REGION and 5’J-REGION of specific combinations of *IGHD-IGHJ* genes; and, (ii) “combinatorial+junctional”, i.e. encoded in part by the N-diversity regions (N1 and N2) leading to restricted motifs at the *IGHV-IGHD* and/or *IGHD-IGHJ* gene junctions
5. the widely shared sequence patterns characteristic of major subsets could cover the entire VH CDR3 or be comprised of a few, even a single, strategically positioned residue; a fascinating example of the latter is subset #2 with 213 *IGHV3-21* expressing CLL cases (2.8% of the cohort) characterized by a very short VH CDR3 sequence (9 amino acids) with a “landmark” amino acid (aspartic acid, D) at position 107, between the amino acids qualified as *IGHV-* or *IGHJ-*encoded (Figure 4)
6. the major stereotyped subsets collectively account for a substantial proportion of the IG CLL repertoire (Figure 5). In more detail, 19 different subsets with 20 or more (up to 213) sequences (defined as major) collectively included 943 cases and, thus, accounted for 41% of the stereotyped cases and 12% of the cohort, respectively. In other words, one-in-eight CLL patients were found to belong to a major subset!

## IG Genes as a Prognostic Marker in CLL: Beyond IGHV Gene Mutational Status

The first hints that not only the mutational status of *IGHV* genes but also other features of the IG BCRs can be prognostically relevant in CLL came in 2002 with the realization that usage of the *IGHV3-21* gene was associated with adverse prognosis independently of the load of SHM –in fact, most *IGHV3-21* rearrangements were mutated.^21^ It did not take long before additional reports were published about genes associated with a distinctive prognosis regardless the actual SHM status -e.g. *IGHV3-23* (adverse),^51^ *IGHV3-30* and *IGHV3-72* (both favorable).^52^,^53^ However, except for the *IGHV3-21* gene, where the supportive evidence is strong,^54^,^55^ these results were obtained in retrospective and mostly small series, hence caution is warranted.

Given how much attraction stereotyped BCRs have held for scientists working on CLL, it was reasonable to explore whether BCR stereotypy might be reflected in stereotyped clinical presentation and outcome. Chiorazzi’s group were the first to report associations between BCR stereotypy and clinical features for a subset of cases expressing stereotyped *IGHV4-39/IGHD6-13/IGHJ5* BCRs (now known as subset #8) who experienced aggressive clinical courses complicated by severe recurrent infections, Richter’s transformation, or the occurrence of second solid tumors.^56^ Of note, a recent collaborative study from Italy independently reported that this particular BCR stereotype is associated with the highest risk for Richter’s transformation among all CLL subgroups analyzed.^57^

A turning point in searching for clinical implications of BCR stereotypy was the realization that among IGHV3-21 CLL those cases carrying stereotyped BCRs typical of subset #2 uniformly expressed CD38 and had progressive disease, whereas cases with heterogeneous IGHV3-21 BCRs exhibited variable CD38 expression and experienced variable clinical courses.^58^ On these grounds, it was suggested for the first time that prognostic information might be gleaned by defining not only the usage of specific genes (e.g. *IGHV3-21*) and their mutational load, but also the molecular features of the BCR IG among cases utilizing similar *IGHV* genes. Since the original publication, several studies have independently shown that expression of stereotyped subset #2 BCRs is associated with shorter time to progression and presence of other poor-prognostic markers.^33^,^36^,^55^

Another clinically relevant example is offered by CLL subset #4 which is defined by the expression of stereotyped IGHV4-34/IGKV2-30 BCRs of the G isotype 59 showing distinctive patterns of SHM.^26^,^60^,^61^ Cases assigned to subset #4 are significantly younger at diagnosis and have been reported to follow a very indolent disease course compared to either the entire cohort or the subgroup of cases expressing non-subset #4 *IGHV4-34* BCRs.^33^

Altogether the available evidence suggests that the biological behavior of CLL malignant B cells, which underlies clonal history and evolution, may be guided by the functional antigen reactivity profile of the BCR. Therefore, the grouping of CLL cases based on shared features of the primary IG gene sequences can be functionally and prognostically relevant.

## Inferring the Role of Antigen in CLL: Hints from Immunogenetic Analysis and Links to BCR Igs With Distinctive Molecular Features

Over the past decade, defining which antigens are recognized by CLL BCRs has been a matter of intense investigation going along with the increasing interest in BCR molecular features as a prognostic indicator for CLL. Earlier studies, from more than two decades ago, had pointed out the autoantigenic and polyreactive nature of CLL-derived monoclonal antibodies (mAbs) (Table 1).^62^-^64^ Major developments came with the advent of new technologies for obtaining the CLL mAbs. Thanks to the new technological possibilities, it was demonstrated that self- and poly- reactivity is inversely correlated with the mutational load of the BCR IG 41 and that CLL mAbs could exhibit common patterns of antigen binding (Table 1).^43^,^45^,^65^

An emerging theme from these antigen reactivity studies is that CLL mAbs with distinctive BCR IG molecular features (i.e. *IGHV* gene usage and mutational status, BCR IG stereotypy) react with molecules present on apoptotic cells, including cytoskeletal proteins, as well as bacterial antigens.^42^,^43^,^45^,^66^ This is similar to the reactivity profile of natural antibodies, which are produced in the absence of external antigenic stimulation and play a crucial role in immediate host defenses against a wide range of pathogens.^67^-^69^

An intriguing finding of these studies was that stereotyped BCR IGs from different subsets showed distinct patterns of antigen reactivity and, more importantly, that CLL mAbs from cases assigned to the same subset exhibited similar (stereotyped) binding profiles.^42^-^45^,^66^ For instance, reactivity against lipid oxidation products created during apoptosis were mainly linked to the unmutated CLL and strong binders of such antigens concerned CLL mABs from stereotyped subsets #1, #6, #8, #9 and #32; on the other hand, vimentin was shown to be recognized by different stereotyped CLL mAbs [*IGHV3-30*, subset #32;^42^ *IGHV4-39*, subset #8 );^45^ *IGHV1-2 or IGHV1-3*, subset #1]^70^ (Table 1).

More recently, Chu et al. showed that non-muscle myosin heavy chain IIA (MYHIIA) expressed on a subpopulation of apoptotic cells (called myosin-exposed apoptotic cells or MEACs) was the antigenic target of CLL mAbs from subset #6 (*IGHV1-69/IGHD3-16/IGHJ3* stereotyped BCRs).^44^ In a subsequent study from the same group, it was shown that MEAC reactivity was not confined to subset #6 mAbs rather it was exhibited by several other CLL mAbs, though variably. MEAC reactivity was far more frequent among unmutated rather than mutated CLL. Of note, mAbs from the same stereotyped subsets showed concordant patterns of MEAC reactivity.^44^,^71^

Self is not the only target of CLL BCRs. Several antigens recognized by CLL mAbs have been functionally associated with certain microbial infections. For instance, molecular mimicry driven by *Streptococcus pneumoniae* capsular polysaccharides and oxidized LDL (oxLDL) has been reported as a critical link between autoreactivity and alloreactivity of CLL mAbs.^72^ Furthermore, certain CLL Abs have been shown to react against various Gram-positive and Gram-negative bacterial strains (*Streptococcus pyogenes, Enterococcus faecium, Enterococcus faecalis, Enterobacter cloacae*); notably, UM-CLL mAbs exhibited the highest level of reactivity, with the unmutated *IGHV1-69* mAbs being the dominant binders.^65^ Perhaps relevant to these observations, a recent epidemiologic study suggested that recurrent respiratory tract infections caused by *Streptococcus pneumoniae* and *Haemophilus influenzae* are associated with an increased risk of CLL (Table 1).^73^

Viral infections have also been suggested to drive subgroups of CLL cases. For instance, persistent infections by EBV and CMV have been correlated with the stereotyped *IGHV4-34* subset #4,^74^ while higher herpesvirus-specific CMV seropositivity has been reported in selected CLL cohorts compared with the general population.^75^ Along these lines, *IGHV1-69* and *IGHV3-21* CLL mAbs have been found to react with the CMV pUL32 protein (Table 1).^76^ Furthermore, the hepatitis C Virus (HCV) has been recently associated with CLL, albeit indirectly. In particular, we showed that the stereotyped *IGHV4-59/IGKV3-20* CLL mAbs of subset #13 can exhibit rheumatoid factor (RF) activity and are linked to HCV infection. Interestingly, we found similar sequences to those of CLL in subset #13 in other entities (including splenic marginal-zone lymphoma, myoepithelial sialadenitis in primary Sjogren’s syndrome, mixed cryoglobulinemia type II and a rheumatoid factor), some with positive HCV serology, others not. The existence of RF with restricted IG gene sequences in various conditions including CLL may allude to cross-reactivity or molecular mimicry of the antigenic elements selecting the clonogenic progenitors yet resulting in distinct pathological conditions.^77^

## BCR- and non-BCR-Mediated Modes of Interaction of CLL Cells with the Microenvironment: a Dynamic and Extensive Cross-Talk

**Figure 6:**
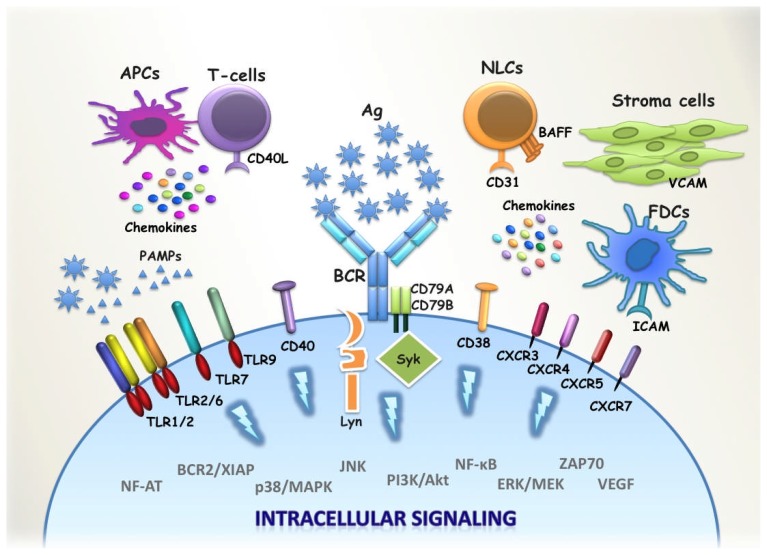
BCR and non-BCR modalities of interactions of CLL cells with their microenvironment The malignant clone is dependent on prosurvival signals conveyed by cell-cell contacts and interactions with soluble factors secreted by T-cells, stromal cells, Nurse-like cells (NLCs), Follicular dendritic cells (FDCs) or other Antigen-Presenting Cells (APCs). The cross-talk between the malignant clone and its millieu is initiated by specific ligand biding to receptor molecules such as the BCR, TLRs, CD38, CD40 and CXCRs. Among the most prominent ligand-receptor interactions are the specific antigenic stimulus of the BCR as well as the triggering of TLRs after recognition of MAMPs. Ligand-Receptor engagement activates a number of different intracellular cascades, which control cell cycle, apoptosis, proliferation and migration.

BCR is of undisputable significance in CLL development and evolution. BCR is not only engaged in antigen recognition but, importantly, it is the start point of intracellular cascades involving downstream kinases, such as Syk, Btk and PI3Kδ (Figure 6).^78^ Interestingly, the kinases involved in BCR signalling have been identified as attractive targets for molecular therapeutic approaches.^79^,^80^

CLL cells preferentially reside in spleen, lymph nodes and bone marrow, where the microenvironment is supportive for expansion since the antigenic stimulus is present together with other promoting signals. Normally, most antigens initiate antibody production by up-regulating CD40L on T cells. CD40/CD40L interaction stimulates B cells to migrate to the germinal center where they undergo SHM and CSR.^81^,^82^ CLL cells have been shown to be generally responsive to CD40 ligation, whereas CD40L stimulation has been shown to up-regulate inhibitor of apoptosis proteins such as survinin.^83^ However, Scielzo et al recently showed that CLL cells exhibit differential patterns of responsiveness upon *in vitro* CD40L stimulation, linked to distinct activation of intracellular signaling pathways (i.e. phoshorylation of IKKα/β, upregulation of BCL2, MCL1) and, intriguingly, to clinical outcome. CD40L-independent cases seem to be less dependent on CD40/CD40L microenvironmental signals, likely because of a higher autonomous proliferative and survival potential and interestingly these cases exhibited a more aggressive disease phenotype.^84^

The crosstalk between CLL cells and components of the adaptive and innate immune system in their microenvironment as well as the exact contribution of the various receptor molecules of CLL cell progenitors or the malignant cells themselves in promoting malignant transformation and progression are still unclear. Recently, we investigated the role of innate immune receptors (Toll-like receptors, TLRs) in CLL (Figure 6). We found that distinct TLR signatures define mutated versus unmutated CLL cases. Furthermore, CLL cases assigned to different subsets with stereotyped BCRs exhibit subset-biased TLR signaling expression profiles.^37^ We also showed that TLRs are functional in CLL cells yet in a heterogeneous fashion and that CLL cases assigned to certain stereotyped subsets exhibit distinct patterns of TLR functionality and/or TLR tolerance. For instance, CLL subsets #1 and #4 do not only differ in disease outcome (subset #1 aggressive, subset #4 indolent) but are also characterized by distinct gene expression profiles of the TLR signaling pathway and distinct responsiveness to TLR ligands.^38^ These findings indicate that CLL cells receive prosurvival signals via both BCR-dependent and -independent pathways, with stereotyped subsets perhaps representing functionally distinct entities with distinct natural histories and patterns of cross-talk with the microenvironment.

## Figures and Tables

**Table 1:** Summary of reactivities reported for CLL.

Study	Antigens involved in CLL	BCR IG	Reference
Bröker BM et al. 1988	IgG-Fc, ssDNA, dsDNA, histones, cardiolipin, cytoskeletal components	unknown	[62]
Sthoeger ZM et al. 1989	ssDNA, dsDNA, rabbit gamma globulin (RGG)	unknown	[63]
Borche L et al. 1990	actin, tubulin and myosin, ssDNA, rabbit gamma globulin (RGG)	IGHV1 and IGHV4 genes Mutation status unknown	[64]
Hervé M et al. 2005	LPS, cytoplasmic structures, DNA, insulin	various	[41]
Lanemo MA et al. 2008	Human tissue samples (e.g. tonsil, stomach chief cells, vascular endothelial cells) FM55M2 melanoma cells, rat aortic smooth muscle cells (SMCs), Jurkat T cells, HepG2 cells, apoptotic Jurkat cells, oxidized low density lipoprotein (oxLDL), cardiolipin, vimentin, filamin B cofilin-1, PRAP-1, S. pneumoniae capsular polysaccharides and phosphorylcholine	various	[42]
Catera R et al. 2008	Healthy HEp-2 cells, apoptotic RAMOS B cells, apoptotic Jurkat T cells Metabolites of lipid peroxidation conjugated to BSA (MDA-BSA, POVPC-BSA, HNE-BSA), Phosphorylcholine (PC)-BSA, oxLDL, tubulin, Sm, Ku, snRNP A, BB’, and C, CENP-B	various	[43]
Chu CC et al. 2008	Non muscle myosin heavy chain IIA (MYHIIA)	*IGHV1-69* unmutated (subset #6)	[45]
Chu CC et al. 2010	MYHIIA-exposed apoptotic cells (MEACs)	*IGHV1-2, IGHV1-3 UM and IGHV1-18* M (subset #1)/*IGHV1-69* UM (subset #6)/*IGHV4-39* UM (subset #8)/*IGHV1-69* and *IGHV3-21* UM (subset#9)/*IGHV1-2 UM* (subset#28)/*IGHV4-b* UM/*IGHV1-3* UM	[44]
Binder M et al. 2010	Vimentin, calreticulin (on viable stroma cells)	*IGHV1-2* and *IGHV1-3* UM (subset #1)	[70]
Hatzi K et al. 2006	Streptococcus pyogenes, Enterococcus faecium, Enterococcus faecalis, Enterobacter cloacae	various	[65]
Landgren O et al. 2007	Streptococcus pneumonia, Haemophilus influenza (population based study)	unknown	[73]
Kostareli E et al. 2009	CMV, EBV (Real Time PCR approach)	*IGHV4-34* M (subset #4)	[74]
Steininger C et al. 2009	CMV seropositivity (population based study )	unknown	[75]
Kostareli et al. 2012	IgG-Fc (with possible link to HCV)	*IGHV4-59* M (subset #13)	[77]
Steininger C et al. 2012	pUL32 protein of CMV	*IGHV1-69* UM, *IGHV3-21* M and UM	[76]
